# Immediate and 6-week effects of wearing a knee sleeve following anterior cruciate ligament reconstruction: a cross-over laboratory and randomised clinical trial

**DOI:** 10.1186/s12891-021-04540-x

**Published:** 2021-08-04

**Authors:** Gisela Sole, Peter Lamb, Todd Pataky, Stefan Klima, Pierre Navarre, Niels Hammer

**Affiliations:** 1 Centre for Health, Activity and Rehabilitation Research (CHARR), School of Physiotherapy, University of Otago, Dunedin, New Zealand; 2 School of Physical Education, Sport and Exercise Sciences, University of Otago, Dunedin, New Zealand; 3 Graduate School of Medicine, Department of Human Health Sciences, Kyoto University, Kyoto, Japan; 4 Orthopaedicus Leipzig, Leipzig, Germany; 5 Orthopaedic Surgeon, Southland Hospital, Invercargill, New Zealand; 6 University of Otago, Dunedin, New Zealand; 7 Department of Macroscopic and Clinical Anatomy, Medical University of Graz, Graz, Austria; 8 Department of Orthopaedic and Trauma Surgery, University of Leipzig, Leipzig, Germany; 9 Fraunhofer IWU, Dresden, Germany

**Keywords:** Anterior cruciate ligament reconstruction, Knee sleeve, Patient reported outcome, Hop distance, Muscle strength

## Abstract

**Background:**

Rehabilitation following anterior cruciate ligament (ACL) reconstructions is based mainly on comprehensive progressive exercise programmes using a multi-dimensional approach. Elastic knee sleeves may be useful adjuncts to rehabilitation. The aim of this study was to determine the immediate and 6-week effects of wearing a knee sleeve on person-reported outcomes and function in participants who had undergone an ACL reconstruction and who had residual self-reported functional limitations.

**Methods:**

Individuals with ACL reconstruction in the previous 6 months to 5 years were recruited. Immediate effects of a commercially-available elastic knee sleeve on single-leg horizontal hop distance were explored using a cross-over design. Following this first session, participants were randomised into a Control Group and a Sleeve Group who wore the sleeve for 6 weeks, at least 1 h daily. Outcome measures for the randomised clinical trial (RCT) were the International Knee Documentation Classification Subjective Knee Form (IKDC-SKF) score, the single-leg horizontal hop distance, and isokinetic quadriceps and hamstring peak torque. Linear mixed models were used to determine random effects. Where both limbs were measured at multiple time points, a random measurement occasion effect nested within participant was used.

**Results:**

Thirty-four individuals (16 women) with ACL reconstruction completed the cross-over trial. Hop distance for the injured side during the sleeve condition increased by 3.6 % (95 % CI 0.4–6.8 %, *p* = 0.025). There was no evidence of differential changes between groups for the IKDC-SKF (Sleeve Group *n* = 15; Control Group *n* = 16; *p* = 0.327), or relative improvement in the injured side compared to the uninjured side for the physical performance measures (Sleeve Group *n* = 12, Control Group *n* = 12; three-way interaction *p* = 0.533 [hop distance], 0.381 [quadriceps isokinetic peak torque], and 0.592 [hamstring isokinetic peak torque]).

**Conclusions:**

Single-leg hop distance of the ACL reconstructed side improved when wearing a knee sleeve. Wearing the knee sleeve over 6 weeks did not lead to enhanced improvements in self-reported knee function, hop distance and thigh muscle strength compared to the control group.

**Trial registration:**

The trial was prospectively registered with the Australia New Zealand Clinical Trials Registry No: ACTRN12618001083280, 28 June 2018.

**Supplementary Information:**

The online version contains supplementary material available at 10.1186/s12891-021-04540-x.

## Background

Anterior cruciate ligament (ACL) ruptures are debilitating knee injuries, potentially with devastating short-term and long-term consequences. Surgical ACL reconstruction and rehabilitation remains the primary approach for active individuals with such ruptures [1]. Reported annual incidence rates per 100,000 person-years for ACL surgeries are 68.6 in the USA [2], 58.2 in New Zealand [3], 52.0 in Australia [4], and 32.0 in Sweden [5]. Different procedures and grafts have been described for the surgical ACL reconstruction. In New Zealand, hamstring tendon grafts account for 71 % of all primary ACL reconstructions, followed by patellar tendon grafts (24 %), and quadriceps tendon grafts (3 %), with allografts used infrequently [6]. Irrespective of graft type, risk of a subsequent ACL rupture ranges between 6 and 15 % [7]. Over 50 % of individuals develop symptoms of knee osteoarthritis within 15 years of reconstruction [8].

Besides the risk of re-injury and knee osteoarthritis, medium to long-term impairments and restrictions following ACL reconstruction have been reported. Cross-sectional studies suggest persistent thigh muscle strength deficits [9, 10], altered movement patterns [11], and lowered levels of physical activity [12] following ACL reconstruction. While muscle strength deficits are dependent on the graft harvest site within 5 or less years following surgery [13, 14], that is less likely in the long term (10 or more years) [15]. Activity levels also appear to decline over time [16]. Long term decreased knee-related quality of life, fear of re-injury and loss of confidence is often experienced following ACL reconstruction [10, 17–19].

Rehabilitation following ACL reconstruction involves multiple elements, such as progressive physical rehabilitation of muscle strength, neuromuscular control, sports- and work-related specific skills and gradual return to physical activity, sports and work [20]. Adjuncts that can be used for rehabilitation following ACL reconstruction are knee braces or sleeves. Historically, rigid knee braces were used as part of early rehabilitation following ACL reconstruction to protect the graft, however, that such braces do not appear to improve clinical outcomes and are no longer prescribed routinely [21, 22]. The use of elastic or neoprene sleeves may be used during rehabilitation post-ACL reconstruction for return to sports [23]. Laboratory studies exploring the efficacy of knee sleeves have focussed on participants with knee osteoarthritis [24] and healthy knees [25]. Results suggest that use of such sleeves may improve function-related performance for symptomatic knees [24, 25], potentially by improving sensorimotor control [26], as well as by improving the individual’s confidence in their knee [25, 27].

The aim of this study was to determine immediate and 6-week effects of wearing a knee sleeve on person-reported outcomes and function in participants who had undergone an ACL reconstruction in the previous 6 months to 5 years, specifically for individuals who had residual self-reported functional limitations. The primary research hypothesis was that single-leg hop distance of the ACL-reconstructed side would improve when wearing a sleeve compared to not wearing the sleeve and compared to the contralateral uninjured side. Secondary hypotheses were that self-reported knee-related symptoms and function would improve to a larger extent over a 6-week period in a group of participants that used the knee sleeve on a daily basis, compared to a control group that did not wear such a sleeve. Lastly, we hypothesised that deficits of the single-leg hop distance and thigh muscle strength would improve to a greater extent for the group wearing the knee sleeve than the control group over the 6-week period.

## Methods

Data were collected during two sessions (baseline and 6-week follow-up) in a University research laboratory and via REDCap (Research Electronic Data Capture, hosted by the University of Otago, Dunedin, New Zealand). CONSORT reporting guidelines were followed [28]. All procedures were performed in accordance to relevant guidelines.

### Trial design and blinding

This study had two linked parts and all participants were involved in both parts. Part 1 consisted of a cross-over laboratory-based study, to examine immediate effects of the wearing of the knee sleeve on single-leg hop distance. Part 2 entailed a parallel two-armed, assessor-blinded randomised clinical trial (RCT), to determine the effects of wearing the knee sleeve over a 6-week period on self-reported knee function and physical performance measures. For the laboratory sessions, it was impossible to blind participants and assessors to the sleeve condition. The research assistant and biostatistician involved in the study were blinded to group allocation for the RCT.

### Participants

#### Recruitment

Participants were recruited via community advertising and using the research participant recruitment agency TrialFacts (https://trialfacts.com/). Volunteers completed a questionnaire (also serving as screening for eligibility) via REDCap prior to attending the first laboratory session. The questionnaire included demographics, injury and surgery history, the International Knee Documentation Committee Subjective Knee Form (IKDC-SKF) [29] and the Tegner activity scale [30]. The Tegner scale categorises sports and physical activity in terms of the level of knee-related loading where ‘0’ indicates ‘sick leave or disability due to a knee injury’ and ‘10’ indicates ‘competitive soccer or rugby at national or international elite level’.

#### Inclusion criteria

We recruited men and women, aged 18–40 years, who underwent ACL reconstruction within 6 months to 5 years previously. We specifically sought individuals who had not yet reached full functional level, defined for the purpose of this study by a score between 40 and 80/100 on the IKDC-SKF [29, 31, 32].

#### Exclusion criteria

Participants were excluded if they had undergone a revision ACL reconstruction of the same knee (due to re-injury), or a previous ACL reconstruction of the opposite knee; self-reported any other lower limb, pelvic or low back musculoskeletal injuries or disorders that required medical care over the past 6 months; had known systemic, neurological or cardiovascular disorders; or had a body mass index (BMI) above 30 kg/m^2^. Participants found to have an IKDC-SKF score less than 40 (due to potential safety risk during the laboratory-based tasks) or greater than 80/100 (as use of a sleeve would clinically be less likely to add benefit) were excluded.

### Procedures

#### Randomisation

Participants were individually randomised twice (once for the cross-over trail, and once for the RCT) with equal numbers in each group for both allocations. Block randomisation (in groups of 8 participants) was undertaken sequentially by a research officer using an electronic random number generator prior to participants being entered into the study. Each group was stratified by sex. The research officer informed the researcher responsible for the laboratory data collection of the order for the conditions for the cross-over trial, and the group allocation (for the RCT) via email prior to the start of the individual participant’s first laboratory session.

 Eligibility to be included was confirmed and participants provided written informed consent at the start of the first session. Participants were asked to be dressed in a singlet, a pair of shorts and their own sport shoes. Body mass and height were measured during the baseline session.

#### Part 1: Laboratory cross-over trial

Participants practised the hopping task at sub-maximal distance with the uninjured and injured sides until they were confident with performing them as part of familiarisation and warm-up. They performed the horizontal hop with the injured side under the (1) ‘control’ condition (no sleeve) and (2) the ‘sleeve’ condition (experimental, wearing the sleeve condition), ordered by randomisation. A 5-minute walk between the conditions provided a standardised run-in to the second condition to minimise carryover effects. On completion of the hopping tasks, the participants underwent the isokinetic thigh muscle strength assessment.

#### Part 2 Randomised clinical trial

Participants were informed of their group allocation for the RCT on completion of the first laboratory session. Following the 6-week period, all participants were asked to return to the laboratory to repeat the above assessments, repeating the hopping tasks (without wearing the knee sleeve) and isokinetic muscle strength tests. Prior to the session, they were sent an electronic REDCap link for the follow-up IKDC-SKF, and they were requested to return their Excel spreadsheet diary to the research officer via email.

### Intervention

The intervention entailed use of a commercially available knee sleeve (company anonymised), a CE-certified medical device. The sleeve consists of flexible elastic/knitted materials to provide support to the knee without restricting the range of motion. For Part 1 (cross-over trial), all participants performed the horizontal single-leg hop with and without the sleeve. For Part 2 (RCT), participants of the ‘Sleeve Group’ (intervention) were instructed to wear the knee sleeve while performing their rehabilitative exercises, physical activity and sports, with a minimum of 1 h per day for the 6-week period; the control group were not provided with a sleeve during this period.

Use of the knee sleeve was explained to the ‘Sleeve group’ participants by the researcher and they were provided an instructional leaflet. They were informed to discontinue use if any side-effects evolved, such as discomfort during use, swelling, pain or burning sensations of the knee, leg, or foot, and to contact a researcher should such complaints arise.

The weekly physical activity may be a confounder for the 6-week outcomes. Thus, participants of both groups were asked to complete a physical activity and exercise diary, documenting the nature, duration and intensity (moderate/hard) of exercises, physical activities and sports involvement over the 6-week period using an Excel spreadsheet (Microsoft Corp, Redmont, WA, USA). Participants of the Sleeve Group were also asked to record the daily duration of wearing the knee sleeve. Participants of both groups still undergoing rehabilitation were encouraged to continue with the programme prescribed by their clinician.

### Outcomes

For Part 1, the primary outcome was the maximal horizontal hop of the injured side and as a deficit compared to the uninjured side. For Part 2, the primary outcome was the IKDC-SKF [29], and secondary outcomes were the maximal horizontal hop, quadriceps and hamstring muscle strength.

#### Horizontal hop

The participant was asked to stand on one leg and to hop as far as they can, landing on that leg (Additional file 1: Appendix 1) [33]. No restrictions were placed on arm movements and participants were asked to hold the landing position for 2 s [34]. If they did not hold the position, the trial was repeated until three successful hops had been performed for each leg and condition. The distance was measured in centimetres from the toe at push-off to the heel on landing. For Part 1, three trials were performed for the injured side without wearing the sleeve and while wearing the sleeve, respectively. The average distance of the three trials for each side and condition were calculated.

#### International Knee Documentation Committee Subjective Knee Form (IKDC-SKF) [29, 31]

This self-report questionnaire consists of 18 questions relating to knee symptoms, function and sports activities. The summed score is on a scale from 1 to 100. Higher scores indicate lower levels of symptoms and higher levels of function and sports activities.

#### Quadriceps and hamstring muscle strength

Quadriceps and hamstring strength was assessed for both sides with an isokinetic dynamometer (Biodex System 3 Pro, Biodex Medical Systems, Inc, Shirley, NY) using previously reported methods [35]. The participant was in a seated position and performed five reciprocal concentric contractions for the knee extensors (quadriceps) and flexors (hamstrings) at 60°/s. The Biodex System 3 DBM (Version 1.7) system software was used to process peak torque for the quadriceps and the hamstrings for the injured and the uninjured sides.

### Sample size

#### Part 1

Given the reported test-retest Intraclass Correlation Coefficient (ICC) of 0.95 for the horizontal hop distance (Additional file 1: Appendix 1 [33]), a conservative correlation was assumed between repeated measures of 0.8 for Part 1. The sample size of 32 participants (all 32 participants receiving both conditions) allowed 80 % power to detect a 0.33 SD difference between the sleeve and no-sleeve conditions using a two-sided test of means at the 0.05 level. This is between a small (0.2 SD) and moderate (0.5 SD) effect size.

#### Part 2

To allow for a weaker correlation between repeated measures of 0.7 and up to 10 % attrition, the sample size would permit a 80 % power to detect differences in changes between the Sleeve Group and the Control Group of 0.86 SD using a two-sided test of means at the 0.05 level, slightly larger than a large effect size (0.8 SD).

### Data analysis

Demographic data were presented descriptively (means and standard deviations for approximately normally distributed continuous variables; geometric means and standard deviations for approximately log-normally distributed continuous variables; medians and interquartile ranges for other continuous variables; and counts and percentages for categorical variables).

Data from the daily exercise/sports diary were expressed in metabolic equivalents (METs) x minutes per week. METs expresses energy cost of physical activities as multiples of metabolic rate [36]. One MET represents an individual’s energy expenditure while sitting quietly and is approximately to 3.5 mL O_2_.kg^− 1^.min^− 1^ (oxygen consumption per kilogram body mass per minute) [36]. The average weekly MET.min were compared between groups, as well as between participants who completed their physical activity during the COVID19 lockdown period and those who were not influenced by the lockdown. Demographic and diary data were analysed using SPSS Version 24.0 (IBM Corp, Armonk, NY).

Hop distance and muscle strength measurements were logarithmically transformed and analyses were adjusted for participant sex, surgery type, and time since surgery (as a continuous measure), and for Part 1 (cross-over trial) only, a sequence effect. Analyses for the IKDC, hop distance and muscle strength are from linear mixed models using Restricted Maximum Likelihood (REML) to estimate random effects. A random participant effect and, where both limbs are measured at multiple time points, a random measurement occasion effect nested within participant. For hop distance, quadriceps and hamstrings peak torque, interaction effects for injured leg with sleeve at follow-up (three-way interaction) and interaction effect for sleeve at follow-up (two-way interaction) are presented as ratios of the geometric means. Reported effects are for changes and, for Part 2, baseline values were incorporated in the model. These analyses were performed with Stata (16.1, StataCorp LLC, College Station, Texas, USA).

## Results

**Fig. 1:**
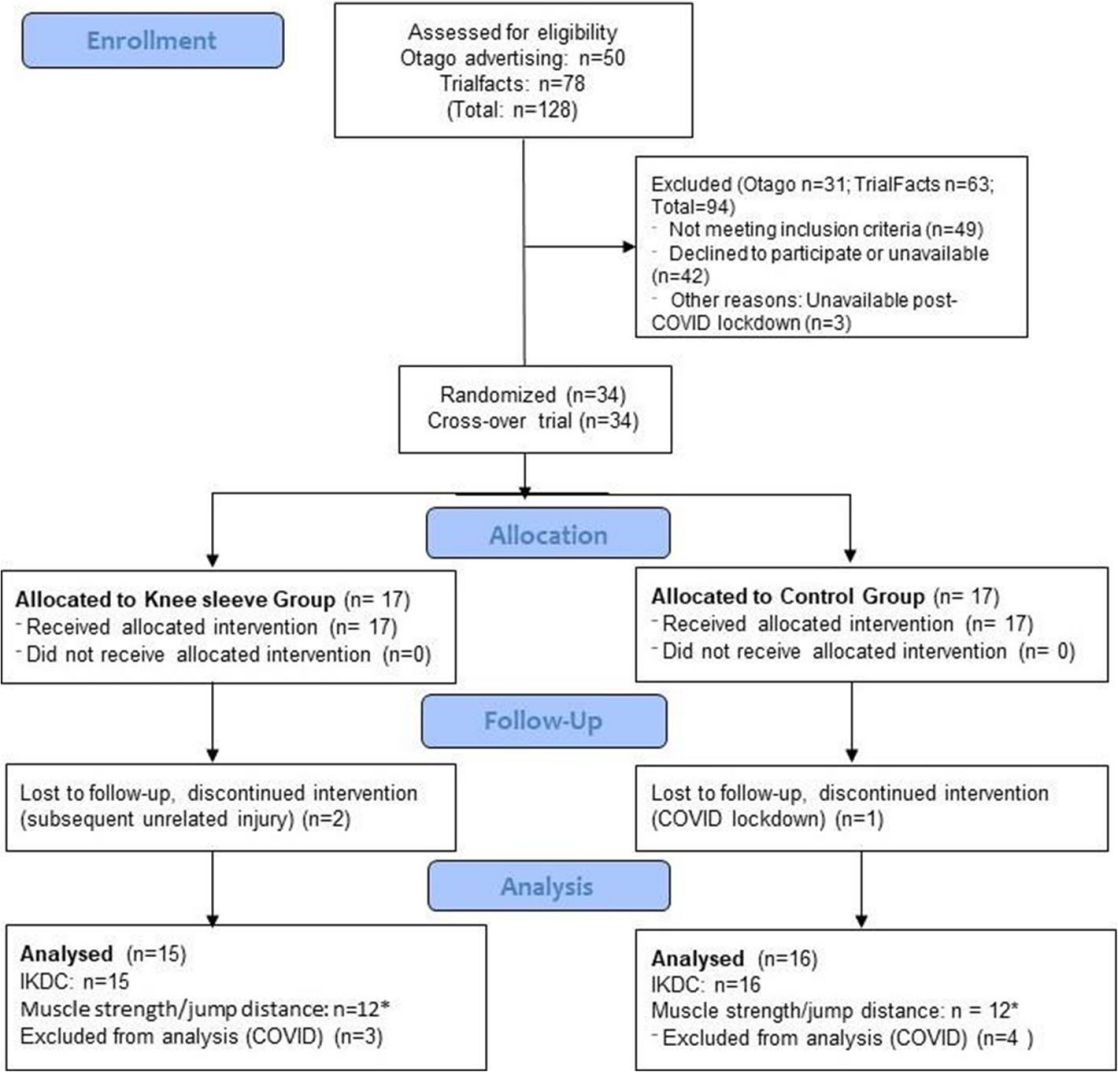
Flowchart of participant recruitment, allocation and follow-up. *Participants were lost to the laboratory-based follow-up data collection due to the COVID-19 lockdown in March/April 2020. IKDC-SKF: International Knee Documentation Committee Subjective Knee Form

One hundred and twenty-eight volunteers responded to community (*n* = 50) and TrialFacts (*n* = 78) advertising. Of those, 34 were eligible, and were assessed at baseline (Part 1). Reasons for exclusion are provided in Additional file 1: Appendix 2. Two participants of the Sleeve Group withdrew from the study following that assessment due to knee re-injuries, unrelated to use of the knee sleeve (Fig. 1). A further eight participants were affected by the COVID-19 lockdown in New Zealand in March/April 2020: one control participant withdrew from the RCT; seven (Sleeve Group *n* = 3; Control Group *n* = 4) continued and completed their physical activity diaries during lockdown. They completed the follow-up IKDC-SKF, but could not attend the second laboratory session. Post-lockdown, recruitment continued and seven participants were included in the study (total *n* = 34). Thirty-one participants completed the follow-up IKDC-SKF (primary outcome for the RCT), of which 24 participants completed the follow-up biomechanical laboratory session (Fig. 1). Demographic data of the participants are provided in Table 1.

**Table 1:** Demographic data (*n* = 34)

	Control Group (*n* = 17)	Sleeve Group (*n* = 17)	*p*-value
Men/Women (n)	8/9	10/7	0.492
Age (Years)	26 (7)	27 (7)	0.504
Mass (kg)	80.4 (11.1)	72.9 (10.7)	0.054
Height (m)	1.73 (0.01)	1.73 (0.10)	0.871
Body mass index (kg.m^− 2^)	26.7 (2.4)	24.4 (3.2)	0.027
Reconstruction: Hamstring/patellar tendon grafts (n)	9/8	7/10	0.492
Meniscal repair: no/yes (n)	13/4	13/4	1.000
Time since ACL injury (months)	21 (9–84)	21 (12–108)	0.849
Time since surgery (months)	16 (6–53)	15 (7–44)	0.809
Time from ACL injury to surgery (months)	6 (1–31)	6 (1–89)	0.958
Tegner activity scale: Preinjury (Median, range)	9 (6–10)	7 (3–10)	0.090
Tegner activity scale: Baseline (Median, range)	5 (3–9)	4 (2–9)	0.661
Self-reported physical activity (MET.min.wk^− 1^) (Median, range, n)	3,070 (488, 6,689; *n* = 12)	7,049 (1,800, 14,347 *n* = 14)	0.009

Numbers show Mean (standard deviations); median (minimum – maximum), or count

*n* number of participants

Tegner score: 0 – disability due to knee problem; 5 – recreational sports, jogging on uneven ground at least twice weekly; 10 – national or international, competitive level team sports (soccer, rugby)

*MET.min.wk*^− 1^ weekly metabolic equivalents and duration (minutes)

### Part 1 Cross-over trial: immediate effects of wearing the knee sleeve

Hop distance increased during the sleeve condition on the injured side by 3.6 % (95 % CI 0.4–6.8 %, *p* = 0.025) (Table 2). The hop distance deficit between the uninjured and the injured side improved from − 9.3 % (-12.4, -6.1 %) without the sleeve to 6.0 % (-9.2, -2.8 %) with the sleeve. This is equivalent to a 5 cm increased performance for the injured limb when wearing the sleeve compared to not wearing the sleeve.

**Table 2:** Hop distance (cm) for the uninjured side and for the injured side (with and without the sleeve) (*n* = 34)

	Mean (SD)	Difference with uninjured (95 % CI)	Difference with injured without sleeve (95 % CI)
Uninjured side	157.0 (36.1)		
Injured side without sleeve	142.6 (32.9)	-9.3 % (-12.4, -6.1)	
Injured side with sleeve	147.2 (32.1)	-6.0 % (-9.2, -2.8)	+ 3.6 % (+ 0.4, + 6.8)

*SD* standard deviation, *CI* confidence intervals

### Part 2 randomised clinical trial

For the IKDC-SKF score, there was no evidence of differential changes between the Control Group and the Sleeve Group (interaction *p* = 0.327) (Table 3). There was no evidence of differences between participants of the Sleeve Group and Control Group in terms of relative improvement in the injured side compared to the uninjured side for the physical performance measures (three-way interaction *p* values were 0.533 [hop distance], 0.381 [quadriceps isokinetic peak torque], and 0.592 [hamstring isokinetic peak torque]) (Table 3). There was no evidence of differences in absolute improvement for the injured side (two-way interaction *p* values for this side were 0.741, 0.060, and 0.338, respectively).

**Table 3:** Means (SD) of physical outcome measures for the Control and Sleeve Groups at Baseline and 6-week Follow-up

Variable	Side	Control Group Mean (SD)		Sleeve Group Mean (SD)		Three-way interaction	Two-way interaction
		Baseline (*n*=17)	Follow-up (*n*=12)	Baseline (*n*=17)	Follow-up (*n*=12)	Mean difference (95% CI), *p* value**	Mean difference (95% CI), *p* value**
IKDC-SKF (/100)^a^		68.0 (8.5)	77.8 (7.9)	67.4 (10.3)	72.1 (14.9)		-3.4 (-10.2, 3.4) 0.327
Hop distance (cm)	Uninjured	154.7 (44.8)	157.7 (46.8)	159.2 (26.1)	165.6 (29.5)	1.04 (0.93, 1.16) 0.533	1.01 (0.93, 1.10) 0.741
	Injured	138.4 (37.4)	141.3 (41.3)	146.8 (28.3)	157.6 (30.0)		
Quadriceps isokinetic peak torque (N.m.kg^-1^)	Uninjured	201.4 (39.4)	215.0 (37.0)	193.7 (42.8)	195.8 (49.3)	0.93 (0.78, 1.10) 0.381	0.89 (0.79, 1.01) 0.060
	Injured	166.5 (44.2)	183.6 (43.6)	164.5 (44.4)	160.7 (49.9)		
Hamstrings isokinetic peak torque (N.m.kg^-1^)	Uninjured	101.5 (24.1)	111.5 (21.6)	100.9 (20.1)	108.0 (25.9)	0.96 (0.82, 1.12) 0.592	0.94 (0.84, 1.06) 0.338
	Injured	88.4 (23.5)	100.5 (18.3)	94.5 (19.1)	100.6 (21.7)		

*SD* standard deviation, *IKDC-SKF* International Knee Documentation Committee Subjective Knee Form

^a^For the IKDC-SKF, sample size for Follow-up was *n*=16 for the Control Group and *n*=15 for the Sleeve Group

**For hop distance, quadriceps and hamstrings peak torque, interaction effects for injured leg with sleeve at follow-up (three-way interaction) and interaction effect for sleeve at follow-up (two-way interaction) are presented as ratios of the geometric means

Twenty-six participants (76 %) returned their physical activity diary. Participants of the Sleeve Group reported higher average weekly METs.minutes than the Control group (Table 1). There was no statistical difference for the weekly METs.minutes between those that completed the trial during the COVID-19 lockdown (*n* = 5; median 6,178 MET.min.wk^− 1^, range 488, 14,347) and the remaining participants (*n* = 21; median 5,041 MET.min.wk^− 1^, range 773, 10,851; *p* = 0.850). Fifteen of the Sleeve Group participants reported wearing the sleeve for a median of 92 min per day (range 42, 434 min).

## Discussion

This study explored whether wearing a knee sleeve had immediate effects on hop distance performance for participants with an ACL reconstruction. The hop distance for the injured side improved by 3.6 % while wearing the sleeve, a 5-cm increase. The deficit, when comparing the hop distance of the injured sides to the uninjured sides, improved by approximately one-third. We also investigated whether a group of such participants wearing the knee sleeve daily for 6 weeks had improved self-reported knee function and physical measures to a greater extent than a control group who was not provided with such sleeve. The results showed that wearing the sleeve did not lead to enhanced improvements in self-reported knee function, hop distance and thigh muscle strength compared to the control group who did not receive that sleeve.

### Participants

The participants had IKDC-SKF scores well below the normative values (85–90) [37] for individuals up to 35 years old, below the defined patient-acceptable state of 85 [38]. The baseline and follow-up scores were similar to previously reported scores for athletes who had not returned to pre-injury levels of sports (73.4, SD 12.3) [39]. The Tegner activity scores ranged from only able to ‘walk on uneven ground’ (2/10) to having returned to competitive team sports (9/10). The diaries showed that most participants were engaged in regular physical activity while two controls did not meet the guidelines for physical activity of at least 1,000 MET.min.wk^− 1^ [36]. Participants of the Sleeve Group reported higher levels of physical activity than the Controls. We do not know whether higher BMI for the Controls (Table 1) suggests that they also had lower levels of physical activity prior to entry into the study. It is possible that being offered a sleeve as part of the trial may have motivated participants of the Sleeve Group to increase their physical activity. However, that remains speculative.

### Immediate effects on hop distance

Single-leg horizontal hop performance may be part of a clinical assessment for patients with ACL reconstruction to assess recovery progress and to determine readiness for return to sports [39, 40]. The findings indicate that a knee sleeve may be useful for people with ACL reconstruction who have residual functional limitations, potentially gaining an immediate small physical improvement. Participants displayed an average hop distance deficit (compared to uninjured sides) ≤ 10 % in this study, which could be considered a successful outcome following reconstruction [41]. However, the distance hopped on the uninjured sides was well below a distance of 187 cm reported for uninjured participants of a similar age group [42]. When interpreting deficits between the injured and uninjured sides, the findings of the latter also need to be considered. Contralateral decreased strength and functional impairments have been described following ACL injury [43–45]. Such changes in the contralateral side are likely due to a combination of central and peripherally mediated mechanisms, as well as lower post-injury levels of physical activity [45, 46]. The mean improvement for the horizontal hop with the sleeve (3.6 %) was marginally greater than a reported standard error of measurement of 3.0 %, and well below a minimal detectable difference of 8 % [34]. Caution is needed in interpretation of these results.

It is unlikely that mechanisms underlying any influences of the knee sleeve are based on mechanical factors due to low mechanical stiffness of the sleeve [24]. As demonstrated in previous laboratory-based studies, wearing a sleeve can enhance knee flexion angles and influence frontal plane biomechanics during walking in participants with knee osteoarthritis [24, 47]. Improvement may also be evident for active joint reposition sense, a proprioceptive variable [25]. One study explored immediate effects of wearing a silicone sleeve in 13 participants within one month of undergoing ACL reconstruction. Passive joint repositioning and isokinetic quadriceps and hamstring muscle strength were reported to be enhanced when compared to no intervention [48]. Collectively, findings indicate that wearing the sleeve may have an influence on sensorimotor control, potentially leading to immediate enhanced movement patterns [24, 26, 47, 48].

### Randomised clinical trial: six-week effects of wearing the sleeve

The lack of significant between-group changes for the IKDC-SKF and the physical measures is likely due to multi-factorial influences for recovery, including injury-related, contextual, physical, and psychosocial influences. With the exception of BMI (and, marginally, body mass), the groups were similar in terms of age, sex ratio, time since injury and reconstruction (Table 1). But a wide range was evident for all outcome measures (Table 2), indicating individual participant variability. Similarly, the self-reported use of the knee sleeve ranged from marginally less than the required average 1-hour per day to wearing the sleeve for close to 8 h per day. Such individual variability and personal contexts may have influenced the between-group comparisons. Overall, our findings indicate that wearing a sleeve is likely not to enhance, nor interfere with recovery within a 6-week period, more than 6 months following ACL reconstruction.

### Clinical implications

There is an increasing global incidence of ACL injury and reconstruction, particularly in young athletes [3, 49–51]. Such injuries reflect significant costs to the individual and health care system, while also placing short-term burden on work- and family-related commitments [52, 53]. Decreased physical activity and increasing body weight following ACL injuries have been noted [54]. Thus, encouraging continued physical activity is required to combat long-term impairments, as well as re-injury. Fear of re-injury and loss of confidence of the knee is frequently reported following ACL reconstruction [41, 55, 56]. Such fear, while a rational response to this injury, may contribute to reluctance in undertaking physical activity and exercise [53, 54, 57]. Interventions are needed to enhance confidence. Graded exposure to functional exercise and sports- and work-specific skills as well as psychologically-informed approaches are used to enhance confidence following ACL injury [58–60]. It is possible that a knee sleeve could be used as an adjunct to such interventions with the aim of enhancing confidence [23, 27]. Based on our results, use of a knee sleeve during rehabilitation can be considered to immediately improve specific activities. However, self-reported knee function, hop distance and thigh muscle strength did not improve to a greater extent for the group wearing the knee sleeve compared to the Control Group at 6 weeks follow-up.

Data from this study thus does not support routine use of knee sleeves in individuals recovering from ACL reconstruction surgery. Further larger studies with longer duration use of sleeves would be required to further assess which type of patients would potentially benefit most from knee sleeves. Based on the results of our study and the current evidence, prescription of knee sleeve as an adjunct to rehabilitation and continued physical activity should be based on individual assessment and response to use of a knee sleeve.

### Methodological considerations

A strength of this study was that we recruited participants with specific self-reported levels of functional limitation, defined by an IKDC-SKF score less than 80/100. While that eligibility criterion challenged our recruitment rate, the strategy enhanced external validity of our findings for ACL reconstruction with residual or persistent restrictions, more than 6 months following ACL reconstruction. Compliance with wearing the knee sleeve, and documenting use as well as physical activity relied on participants’ self-report. Use of pedometers, mobile physical activity apps and wearable technology may have given greater confidence in those results, however, those devices are also dependent on participants choosing to use them [61]. In this study, both groups used the same format for the diaries and were sent email reminders by a research team member.

This study was affected by the 6-week COVID-19 lockdown of 2020 in New Zealand, loosing 8 participants for the laboratory follow-up session. Despite continuing recruitment post-lockdown, we were unable to recruit sufficient participants to meet the planned sample size of 16 per group for the RCT within the funding period. Our results may thus reflect a Type 2 error for the 6-week effects of wearing a sleeve. Our sample size calculation was based primarily on the cross-over trial, and not the RCT, further adding towards risk of type II error rate for the latter. Finally, due to research staff changes during the course of the study, the researcher collecting data was not blinded to the group allocation for the RCT. Standard instructions were provided to the participants for the hop test and the muscle strength test. Hop test distance was measured by a research assistant blinded to the group allocation. Isokinetic peak torque was processed and extracted using the Biodex software, thus was not influenced by lack of blinding.

## Conclusions

In a group of 34 participants with ACL reconstruction, single leg hop distance on the injured side improved immediately when wearing a knee sleeve compared to not wearing the sleeve. However, wearing the sleeve for 6 weeks for a at least 1 h per day did not lead to enhanced improvements in self-reported knee function, hop distance and thigh muscle strength compared to the control group who did not receive that sleeve.

## Supplementary Information

**Additional file 1: Appendix 1.**Description and psychometric properties of outcome measures. **Appendix 2.** Reasons for exclusion from the study

## Data Availability

The datasets used and/or analysed during the current study are available from the corresponding author on reasonable request.

## Abbreviations

ACL: Anterior cruciate ligament.
BMI: Body mass index.
IKDC-SKF: International knee Documentation Classification Subjective Knee Form.
METs: Metabolic equivalents.
RCT: Randomised clinical trial.
