# Supplementary material for: Immediate and 6-week effects of wearing a knee sleeve following anterior cruciate ligament reconstruction: a cross-over laboratory and randomised clinical trial
Source: BMC Musculoskelet Disord. 2021 Aug 4;22:655. doi: 10.1186/s12891-021-04540-x (PMC8336666; doi:10.1186/s12891-021-04540-x)
Supplement: Supplementary file 1 — Additional file 1: Appendix 1.Description and psychometric properties of outcome measures. Appendix 2. Reasons for exclusion from the study [file 12891_2021_4540_MOESM1_ESM.docx]

**Appendix 1: Description and psychometric properties of outcome measures**

| Outcome measure | Additional description and psychometric properties |
| --- | --- |
| Single-leg horizontal hop distance | Typical Limb Symmetry Index means (SD) following ACL reconstruction (22 weeks postoperative) are 88.2% (9.5) [1]. That translates to a deficit of 11.8%. In general, a deficit ≤10% is defined as a guideline as part of testing batteries to assess readiness for return to sport [2]. ICCs of absolute hop distance (in cm) have been reported to be 0.95 for men and women following ACL reconstruction [3]. SEM of 3.0% and minimal detectable difference of 8% has been defined [1]. |
| International Knee Documentation Committee Subjective Knee Form (IKDC-SKF) [4, 5] | A patient acceptable symptom state (PASS, reflecting an acceptable state that corresponds to ‘feeling well’) of 85/100 (sensitivity 0.83; specificity 0.96) has been reported for individuals with ACLR, up to 5 years post-surgery [6]. An ICC of 0.93 (95% CI 0.89-0.96) and SEM of 4.4 have been reported [5]. The minimal important clinical difference (MICD) is unclear for ACL reconstruction, but has been reported to be 6.3 at 6 months and 16.7 at 12 months following knee cartilage repair [7, 8]. A MICD of 10 was applied for the purpose of this study. |
| Thigh muscle peak torque | Test-retest reliability of peak torque during these muscle contractions were above 0.90. SEMs between 5-10% and smallest detectable differences of 12 – 25% for peak torque have been reported [9]. |
| ACL: anterior cruciate ligament; ICC: Intraclass correlation coefficient; SD: standard deviation; SEM: standard error of measurement | |

**Appendix 1 Reference list**

1. Reid A, Birmingham TB, Stratford PW, Alcock GK, Giffin JR: Hop testing provides a reliable and valid outcome measure during rehabilitation after anterior cruciate ligament reconstruction. *Phys Ther* 2007, 87(3):337-349.

2. Edwards PK, Ebert JR, Joss B, Ackland T, Annear P, Buelow J-U, Hewitt B: Patient characteristics and predictors of return to sport at 12 months after anterior cruciate ligament reconstruction: The importance of patient age and postoperative rehabilitation. *Orthop J Sports Med* 2018, 6(9):2325967118797575.

3. Gustavsson A, Neeter C, Thomee P, Silbernagel KG, Augustsson J, Thomee R, Karlsson J: A test battery for evaluating hop performance in patients with an ACL injury and patients who have undergone ACL reconstruction. *Knee Surg Sports Traumatol Arthrosc* 2006, 14(8):778-788.

4. Irrgang JJ, Anderson AF, Boland AL, Harner CD, Kurosaka M, Neyret P, Richmond JC, Shelborne KD: Development and validation of the international knee documentation committee subjective knee form. *Am J Sports Med* 2001, 29(5):600-613.

5. van Meer BL, Meuffels DE, Vissers MM, Bierma-Zeinstra SMA, Verhaar JAN, Terwee CB, Reijman M: Knee Injury and Osteoarthritis Outcome Score or International Knee Documentation Committee Subjective Knee Form: Which questionnaire is most useful to monitor patients with an anterior cruciate ligament rupture in the short term? *Arthroscopy* 2013, 29(4):701-715.

6. Muller B, Yabroudi MA, Lynch A, Lai C-L, van Dijk CN, Fu FH, Irrgang JJ: Defining thresholds for the Patient Acceptable Symptom State for the IKDC Subjective Knee Form and KOOS for patients who underwent acl reconstruction. *Am J Sports Med* 2016, 44(11):2820-2826.

7. Collins NJ, Misra D, Felson DT, Crossley KM, Roos EM: Measures of knee function: International Knee Documentation Committee (IKDC) Subjective Knee Evaluation Form, Knee Injury and Osteoarthritis Outcome Score (KOOS), Knee Injury and Osteoarthritis Outcome Score Physical Function Short Form (KOOS-PS), Knee Outcome Survey Activities of Daily Living Scale (KOS-ADL), Lysholm Knee Scoring Scale, Oxford Knee Score (OKS), Western Ontario and McMaster Universities Osteoarthritis Index (WOMAC), Activity Rating Scale (ARS), and Tegner Activity Score (TAS). *Arthritis Care Res* 2011, 63 Suppl 11:S208-228.

8. Greco NJ, Anderson AF, Mann BJ, Cole BJ, Farr J, Nissen CW, Irrgang JJ: Responsiveness of the International Knee Documentation Committee Subjective Knee Form in comparison to the Western Ontario and McMaster Universities Osteoarthritis Index, modified Cincinnati Knee Rating System, and Short Form 36 in patients with focal articular cartilage defects. *Am J Sports Med* 2010, 38(5):891-902.

9. Sole G, Hamren J, Milosavljevic S, Nicholson H, Sullivan SJ: Test-retest reliability of isokinetic knee extension and flexion. *Arch Phys Med Rehabil* 2007, 88(5):626-631.

**Appendix 2: Reasons for exclusion from the study**

| Criteria | Number* | Percentage of 49 volunteers |
| --- | --- | --- |
| Not able to attend site (geographically) | 9 | 18% |
| Body Mass Index (> 30 kg.m^-2^) | 16 | 32% |
| Age (not between 18 and 40) | 3 | 6% |
| Did not have a reconstruction | 7 | 14% |
| International Knee Documentation Committee (IKDC, not <80/100) | 6 | 12% |
| Bilateral ACL reconstructions | 8 | 16% |
| More than one anterior cruciate ligament reconstruction on same side | 17 | 34% |
| Pregnant | 2 | 4% |
| Currently have or had any other lower limb, pelvic, or lower back injuries in the last 6 months | 11 | 22% |

*Total number is more than 49 as more than one exclusion criteria may have applied per volunteer
